# How emergency digital health and data use investments can strengthen health systems and support global health security

**DOI:** 10.1093/oodh/oqae004

**Published:** 2024-05-06

**Authors:** Amarynth Sichel, Adele Waugaman, Robert Rosenbaum, Joy Kamunyori, Emily Lark Harris, Marc Cunningham, Folake Olayinka, Beth Tritter

**Affiliations:** Bureau for Global Health, United States Agency for International Development, GHTASC-PHI, 500 D Street SW, Washington, DC, 20024, USA; Bureau for Global Health, United States Agency for International Development, GHTASC-PHI, 500 D Street SW, Washington, DC, 20024, USA; Bureau for Global Health, United States Agency for International Development, GHTASC-Credence, 500 D Street SW, Washington, DC, 20024, USA; Bureau for Global Health, United States Agency for International Development, GHTASC-Credence, 500 D Street SW, Washington, DC, 20024, USA; Bureau for Global Health, United States Agency for International Development, 500 D Street SW, Washington, DC, 20024, USA; Bureau for Global Health, United States Agency for International Development, GHTASC-PHI, 500 D Street SW, Washington, DC, 20024, USA; Bureau for Global Health, United States Agency for International Development, GHTASC-PHI, 500 D Street SW, Washington, DC, 20024, USA; Bureau for Global Health, United States Agency for International Development, 500 D Street SW, Washington, DC, 20024, USA

**Keywords:** digital health, data use, resilience, global health security, COVID-19, digital health enabling environment

## Abstract

This commentary is focused on a body of research examining a subset of the United States Agency for International Development (USAID) global vaccine delivery investments made during the emergency phase of the coronavirus disease 2019 pandemic (COVID-19). Taken together, the research illustrates the importance of the digital health enabling environment—the presence or absence of conditions that allow digital health investments to thrive—as a contributor to an effective emergency response and as a determinant of whether digital investments deployed during an emergency can contribute to stronger health systems and increased preparedness for future health shocks. The commentary distills findings from this journal supplement into three high-level insights and offers recommendations to translate these insights into actions that can improve future health emergency responses and strengthen health systems.

Abrégé

Ce commentaire se concentre sur un corpus de recherche examinant un sous-ensemble des investissements mondiaux de l’USAID dans la livraison de vaccins réalisés pendant la phase d’urgence de la pandémie de COVID-19. Prise dans son ensemble, la recherche illustre l’importance de l’environnement propice à la santé numérique – la présence ou l’absence de conditions permettant aux investissements en santé numérique de prospérer – en tant que facteur contribuant à une intervention d’urgence efficace et permettant de déterminer si les investissements dans le numérique réalisés pendant une urgence peuvent jouer un rôle positif dans le renforcement des systèmes de santé et l’amélioration de la préparation aux futurs chocs sanitaires. Le commentaire tire des conclusions présentées dans ce supplément de revue trois enseignements de haut niveau et formule un certain nombre de recommandations pour traduire ces enseignements en actions susceptibles d’améliorer les futures interventions d'urgence sanitaire et de renforcer les systèmes de santé.

Resumen

Este comentario se centra en un corpus de investigaciones sobre un subconjunto de las inversiones que USAID realizó para la entrega de vacunas en todo el mundo durante la fase de emergencia de la pandemia de COVID-19. En conjunto, las investigaciones demuestran la importancia del entorno propicio para la salud digital, es decir, la presencia o ausencia de condiciones que permitan que las inversiones en salud digital prosperen, como factor que contribuye a una respuesta de emergencia efectiva y como determinante de si las inversiones digitales implementadas durante una emergencia pueden contribuir a fortalecer los sistemas de salud y aumentar la preparación para futuras crisis de salud. El comentario resume los hallazgos de este suplemento de revista en tres ideas de alto nivel y ofrece recomendaciones para traducir estas ideas en acciones que puedan mejorar las futuras respuestas a emergencias sanitarias y fortalecer los sistemas de salud.

## INTRODUCTION

This supplement is dedicated to taking stock of learnings from the use of digital technologies and data systems (also referred to as digital health) during the global effort of the United States Agency for International Development (USAID) to deliver coronavirus disease 2019 (COVID-19) vaccines. Digital tools can facilitate timely access to data, which is vital for managing an effective health emergency response. However, without strategic planning and coordination, the broader system strengthening impact of these tools can be limited, despite millions of dollars of investment.

The research examines how digital health investments were made as part of USAID’s COVID-19 vaccine delivery effort and helps answer three questions. First, are there indications that digital health investments made to address emergency needs can contribute beyond the crisis response to strengthen health systems? Second, what factors can support or inhibit this system strengthening effect? Third, how can such investments contribute toward global health security by bolstering country health emergency preparedness and response capabilities? The answers to these questions provide insight into how to strengthen global health security and how to ensure that investments in country digital and data systems can yield the broadest possible value, recognizing that health systems may be under increasing strain as pandemics become more likely and as climate change increases zoonotic disease risks [1, 2]. The findings from this supplement provide actionable lessons from the COVID-19 pandemic response to shape subsequent global health investments.

Severe acute respiratory syndrome coronavirus 2, the strain of coronavirus that caused the COVID-19 pandemic, challenged the existing global health infrastructure in unprecedented ways. The novel disease required rapid development of clinical guidance, prevention and treatment protocols, and outbreak response planning. The geographic scope and severity of the pandemic strained health system resources and capacity around the world. Most notably, the disease took a heavy toll on human life, particularly among the elderly, people with comorbidities, and communities facing inequities.

From a digital systems perspective, the global COVID-19 vaccine delivery effort presented both familiar and novel challenges. As in the past, the emergency response had to overcome hurdles related to gaps in power and connectivity infrastructure, foundational country-level health datasets and the scale and interoperability of digital systems. Insufficient connectivity confounded efforts to mobilize, reach and follow up with priority populations, particularly in remote areas. Gaps in public health data, such as accurate population denominators, health facility geolocation and up-to-date health worker registries, further challenged the ability of all actors to mount a swift and effective response. Some countries had duplicative digital systems that were not interoperable, such as those managing the supply chains of different health commodities, and lacked systems scaled to track nationwide vaccine delivery. COVID-19 introduced new data needs that presented challenges for existing digital systems. Where digital supply chain systems did exist, for example, few were prepared to manage the ultra-cold chain data tracking required to safely transport and store mRNA vaccines. Where electronic immunization registry systems were in place to manage vaccine administration data, these systems frequently lacked the ability to track multi-course immunization schedules across age groups.

Similar challenges were documented by USAID in the stock-taking of international efforts to leverage data, information and digital technologies during the 2014–16 West Africa Ebola outbreak response [3]. USAID acted on those lessons by incorporating related recommendations into policy guidance through its ‘Vision for Action in Digital Health’, which identified four priorities to guide future USAID digital health investments [4]. These priorities are to support and strengthen (1) a country’s digital health enabling environment, (2) national digital health strategies and (3) national digital health architecture, including through leveraging (4) global goods, where appropriate [5].

The studies in this supplement offer examples of how organizations funded by USAID to meet COVID-19 vaccine delivery needs considered these priorities as part of their digital and data-related efforts. Their work spanned 11 countries in Africa, Asia and Central and South America: Burkina Faso, the Democratic Republic of the Congo, Honduras, Indonesia, Kenya, Mali, Niger, Senegal, Suriname, Tanzania and Vietnam. The research examined to what extent, and how, their activities leveraged and contributed to country digital systems and capacity that could bolster preparedness and strengthen health systems.

## DIGITAL TECHNOLOGY AND DATA SYSTEMS USE IN COVID-19 VACCINE DELIVERY

COVID-19 presented an unprecedented global challenge: to vaccinate a broad swath of the world’s population while fully covering the highest risk groups on a compressed timeline. For USAID, this required a large-scale rapid response, mobilizing $10 billion to combat the virus in low- and middle-income countries and coordinating with actors in the US government, USAID's partner country governments, other donor governments, a variety of multilateral and regional organizations, development banks, the private sector, and civil society.

USAID’s planning for and delivery of over 680 million COVID-19 vaccines to partner countries relied on an array of digital health and data systems, tools and services that supported information needs such as [6, 7]:

- Planning, including the identification of high-risk populations for vaccination
- Pre-registration, allowing individuals to signal interest in vaccination, schedule an appointment and receive appointment reminders
- Supply chain and logistics management systems that tracked COVID-19 vaccines and other commodities
- Records management, linking individuals with information about their vaccinations and enabling health officials to track vaccine coverage
- Vaccine certificates for individuals, an important record during a time of limited mobility without proof of vaccination
- Monitoring systems, including to track adverse effects following immunization
- Community engagement to enable coordinated, interactive information-sharing with the public
- Human resources for health training, supportive supervision and management

The impact of COVID-19 exceeded the bounds of the World Health Organization (WHO)-declared Public Health Emergency of International Concern phase of the pandemic, underscoring the importance of digital systems and capacity investments that can outlast the emergency phase of a global health response. Lingering impacts of the COVID-19 pandemic include depressed global life expectancy, the reversal of health gains across areas including childhood immunization and the fight against tuberculosis and HIV, and the burden that the continued transmission of new COVID-19 variants of concern places on health systems.

## ABOUT THIS RESEARCH

Monitoring and evaluation plans associated with the activities profiled in this supplement explored the impact of COVID-19 vaccination efforts; however, to our knowledge, there was no body of research dedicated to understanding whether and how emergency digital health investments to support COVID-19 vaccination efforts contributed toward other health areas and to more resilient health systems over medium- and long-term time horizons, respectively. This research sought to fill that gap, with a focus on medium-term outcomes, as it may be too early to evaluate long-term system-level effects.

The activities included in this review were drawn from the work of four mechanisms funded by USAID’s Bureau for Global Health to provide digital health and data use implementation support, as part of USAID’s more than $1.23 billion contribution to the US Government’s Initiative for Global Vaccine Access. These mechanisms—Country Health Information Systems and Data Use, Data for Implementation, Digital Square and MOMENTUM-Routine Immunization Transformation and Equity—received approximately $8 million combined to carry out the work profiled in this supplement from the USAID Bureau for Global Health and country missions. The Global Health Bureau collaborated with regional bureau colleagues to identify needs and avoid duplication of efforts. Funded activities began around March 2022 and included a range of interventions focused on improving data collection, information systems, processes and data quality, analysis, and use [8]. Research approaches varied by project, leveraging a mix of qualitative and quantitative methods, including key informant interviews, focus group discussions, direct observation, and stock and aggregate health data analyses.

The activities profiled in this research represent a limited subset of USAID support for COVID-19 response-related digital health needs in two respects. First, the activities focused only on vaccination efforts as opposed to disease surveillance, treatment, or other aspects of the COVID-19 response. Second, they reflect only a subset of USAID's COVID-19 vaccination funding and do not include a multitude of activities funded by USAID country offices. However, what the scope of this research lacks in breadth, we hope will be balanced through depth, thoughtfully probing whether or how these activities benefited from or contributed to a country’s digital health enabling environment.

We are publishing these findings after the global emergency vaccination drive is over, but before longer-term or systems-level outcomes can be realized or measured. We share these insights now because we believe they can inform how future emergency response funding is leveraged to both strengthen systems and address emergency needs.

## KEY FINDINGS

**Figure 1:**
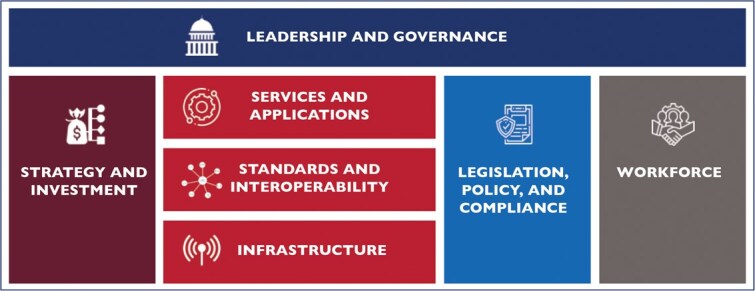
A country’s digital health enabling environment is commonly understood to refer to seven building blocks: country digital health leadership and governance; strategy and investment; infrastructure; services and applications; standards and interoperability; legislation, policy, and compliance; and workforce [5, 8, 9].

Three primary findings emerge from this research. Taken together, these studies point unequivocally to the importance of the digital health enabling environment (Fig. 1) as a key contributor to effective emergency response and as a determinant of whether digital investments outlive the crisis in which they were deployed to provide enduring value.

### Digital health investments designed to support COVID-19 vaccination efforts added value beyond the emergency phase of the response

A major line of inquiry throughout the studies in this supplement explored whether digital health investments made to meet needs of the COVID-19 vaccination effort could contribute toward broader health system strengthening. The theory of change developed to organize this research illustrates the hypothesis that if digital interventions contributed toward strengthening the building blocks of the enabling environment, there might be health system strengthening effects [8, Figure 1]. Evidence from this supplement shows that emergency digital health investments can support other health areas beyond the emergency response, particularly when interventions are designed with this approach in mind. Although there was insufficient time to explore longer-term health system impacts, the institutionalization of COVID-19 digital health vaccine investments to support other health areas provides encouraging evidence that these investments continue to offer value and may contribute toward health system strengthening.

For example, the Honduras Ministry of Health adopted an intervention to strengthen coordination and data use through a situation room approach, which brought together a cross-sectoral leadership team for structured and sustained data review and coordination to bolster the country’s COVID-19 response. This practice resulted in self-reported improvements in leadership use of data to make evidence-based decisions for the COVID-19 response. The situation room approach is already being adapted in Honduras to address other health areas, including tuberculosis, influenza, mpox and maternal mortality. In Burkina Faso, digital tools that were implemented to track COVID-19 vaccination data at the individual (versus aggregate) level are being used to support other health areas, such as malaria and cervical cancer. In Mali, a maturity assessment conducted to understand interoperability potential for digital tools to support the COVID-19 emergency response is informing enterprise architecture decisions. In Indonesia, a mobile application with over 100 million users that served as a digital vaccine certificate and a tool for contact tracing is being expanded to serve as a mobile personal health record.

### Prior investment in a country’s digital health enabling environment helped emergency investments contribute to an effective pandemic response and support other health areas, beyond the COVID-19 response

Prior investment in a country’s digital health enabling environment helped facilitate the emergency COVID-19 vaccination response. For example, following the 2014 to 2016 Ebola outbreak in West Africa, USAID supported a One Health activity in Burkina Faso that invested in an interoperability layer to facilitate data exchange between the ministries of health, environment, and animal resources. During the COVID-19 vaccination response, Burkina Faso’s Ministry of Health leveraged the One Health database to deploy a COVID-19 data system for case management, contact tracing, testing, mortality tracking, and, eventually, vaccination tracking. The data-sharing facilitated by this interoperability layer, which allowed for data exchange between the ministry's COVID-19 vaccine tracker, lab information system, and the systems that provided public access to COVID-19 test results and vaccination certificates, also enabled health workers to efficiently deliver COVID-19 test results and vaccination cards. The prior investment in interoperability, which was aimed at strengthening disease surveillance and outbreak response capacity, provided a strong and flexible digital foundation that the Ministry of Health leveraged to quickly address COVID-19 health needs as they emerged.

This supplement also offers evidence indicating that previous enabling environment investments can help emergency systems add value beyond the pandemic response. For example, prior to and during the pandemic, Indonesia invested in interoperability through a national health information exchange platform. During the COVID-19 vaccination campaign, the Ministry of Health developed a mobile application (SATUSEHAT Mobile) for digital vaccine certificates and contact tracing that was interoperable with the national health information exchange platform. SATEUSEHAT Mobile is now being expanded to serve as a mobile personal health record, which relies on the national exchange platform to access and share health data. The investment in interoperability via the information exchange platform made it easier for the Ministry of Health to expand the use of SATUSEHAT Mobile beyond COVID-19 to support routine immunization with plans for further expansion to maternal and child health, tuberculosis, and other health areas. As referenced above, SATUSEHAT Mobile had over 100 million users at the time of this paper's drafting.

Studies in this supplement also highlight where gaps in the digital health enabling environment, such as around infrastructure and workforce, hindered the pandemic response. For example, lacking connectivity infrastructure contributed to data backlogs in Kenya, Senegal, and Tanzania. As in previous health emergencies, health workforce gaps, including worker shortages, high workloads and lack of payment, negatively impacted health workers and contributed to slowed information flows. Data backlogs stemming from infrastructure and health workforce challenges created significant time lags (often many months long) in governments' ability to understand precisely who had been vaccinated and where coverage gaps persisted among high-risk, priority populations. In addition, national digital health strategies and architecture were nascent or absent in many countries. A paucity of investment into these fundamental pillars of country digital health maturity contributed to a lack of clarity about what resources were available or required to most strategically align emergency funds to meet country needs.

### Stopgap measures were important early in the COVID-19 vaccination effort but created their own challenges later in the response and did not support health system strengthening

To facilitate a rapid response, many countries implemented stopgap measures that they could deploy quickly. For example, in countries including the Democratic Republic of the Congo, Kenya, and Mali, there was a proliferation of data systems deployed to support COVID-19 vaccination efforts, from notebooks to Excel spreadsheets. These stopgap measures helped enable data collection during campaigns early in the vaccination effort when countries lacked more robust data systems, and when vaccine supply was more limited. The data they collected were important for ensuring that vaccination efforts were reaching high-risk populations, such as frontline workers and the elderly. However, while quick to deploy, over time such tools generated their own obstacles: they typically did not allow multiple users to enter data simultaneously, had limitations in the amount of data they could process, and had data access challenges, including security risks. Quick stopgap solutions may be an appropriate—and even vital—strategy in the early phase of an emergency response; however, over time these approaches were not sustainable and contributed to vaccination data bottlenecks, which impeded vaccination efforts.

Countries also faced trade-offs between prioritizing aggregate versus individual-level data. Collecting aggregate data was quicker, often more familiar to frontline workers, and required fewer digital data-entry devices. Collecting individual-level data provided decision-makers with more precision about the reach of vaccination efforts. In both instances, the key to transitioning from more basic (e.g. paper or spreadsheet-based) data collection to more sophisticated digital tools relied on the initial tool being designed with future enhancements—whether in the digital software used or the granularity of data collected—in mind. Where such transitions were not anticipated and planned for, challenges often stymied plans to integrate systems and improve overall performance.

## RECOMMENDATIONS

The following recommendations translate these findings into actions that health system planners, funders and implementers can take now to improve future health emergency responses and contribute toward strengthening health systems. These recommendations are also relevant for non-health system country leaders. Experience with the COVID-19 pandemic response underscored that in health emergencies, decision-making power may reside with leaders in other areas of government, including the executive branch.

### Understand and invest in the digital health enabling environment

Evidence from this supplement illustrates that the strength of a country’s digital health enabling environment can support an effective pandemic response and help emergency-era digital health investments contribute to the broader health system. Investing in the enabling environment should be considered core to routine health programs and to pandemic preparedness and response efforts.

#### Incorporate regular digital health enabling environment assessments into country and health program planning processes

Understanding the enabling environment, including the digital systems in use, can help decision-makers adapt and scale existing systems to meet new health emergency-related needs. This awareness can also help decision-makers coordinate emergency investments to leverage the strengths of the enabling environment and address gaps that may undermine an emergency response. Enabling environment assessments should be informed by national digital health strategies, architectures and costed implementation roadmaps where they exist. Assessments should identify needed investments aligned to these critical pillars of the enabling environment. Where national strategies, architecture and costed implementation roadmaps are absent or nascent, assessments can help chart a course to develop or strengthen them.

#### Invest in interoperability

A key component of disease outbreak preparedness includes thinking beyond individual health programs to build interoperable systems that can bring together operational and epidemiological data to inform all phases of a health emergency response.

#### Build solutions to support the health workforce now and during the next outbreak response

This includes systems that can send health workers direct payments. By facilitating compensation, such solutions can help address preventable health worker morale challenges before they can impact a health emergency response. Other solutions include investing in human resource systems to keep track of trained health workers from one health emergency to the next and to help address workforce shortages.

#### Develop partnerships with the private sector to bridge infrastructure and funding gaps

This can include pre-arranged agreements with telecom providers to support health workers with data bundles during a health emergency.

#### Provide guidance to health program implementers on how to factor enabling environment considerations into emergency planning

Not all partners implementing digital solutions during a health emergency will be familiar with the digital health enabling environment or have digital health expertise. To help implementers design digital health activities that address urgent needs in a way that can contribute to system strengthening, funders can offer light capacity strengthening before an outbreak occurs, develop a checklist outlining potential actions to strengthen the enabling environment and require that activity plans incorporate environment strengthening considerations.

### Develop a suite of data standards and guidelines to support decision-makers at all levels in an outbreak response

#### Develop standards outlining data fields to be collected, levels of disaggregation and digital system requirements that can be adapted based on the type of disease outbreak and the implementation context

The WHO, other standards-making bodies and others supporting global health security should define standard outbreak response indicators to help support congruity between data collection systems and facilitate consolidation in the event that multiple data systems are used, including paper systems. All standards and guidance should be provided in a software-agnostic format and offer guidance around suggested levels of data disaggregation that can be adapted based on different levels of country enabling environment maturity. Such guidance can support countries in making rapid judgment calls about how to prioritize the ease of aggregate data collection against the increased precision of disaggregated data collection.

#### When employing stopgap solutions, develop transition plans to move toward more robust systems, with consideration for change management

Plan for the capacities or tools necessary to facilitate the transition away from stopgap measures toward solutions that enable greater usability and functionality. For example, consider capacity strengthening for health emergency decision-makers focused on software system requirements gathering, or developing toolkits to guide data system review and redesign processes. Ideally, the transition away from stopgap solutions would not require maintaining parallel systems for long, as this can contribute to data backlogs.

### Support further research to explore the relationship between strengthening the digital health enabling environment and broader health system strengthening

#### Support further inquiry to understand with more granularity whether, and under what conditions, the connection between these two elements holds true

This research hypothesizes that investments that leverage and strengthen the digital health enabling environment will contribute to strengthening health systems [8, 9]. However, due to the timeframe of this research, long-term health system outcomes could not be directly assessed. The evidence illustrating that COVID-19 digital health investments contributed to bolstering elements of the digital health enabling environment, such as services and applications, and were institutionalized to support other areas within health systems offers preliminary support for this hypothesis, but further research should explore and clarify these connections.

#### Develop standardized measures to take stock of how strengthening the enabling environment advances health system performance

The Global Digital Health Monitor has emerged as a standard for measuring countries’ digital health enabling environments. In some countries, it is being used to support national digital health strategy planning and implementation tracking [10]. This approach could be deepened to measure the impact of the digital health enabling environment on the availability, quality and timeliness of data as well as its impact on the intermediate outcomes of health system strengthening, including equity, quality and resource optimization [11].

## CONCLUSION

In a resource-constrained world with complex health challenges, orienting emergency funds both to meet immediate needs and strengthen health systems can achieve the dual objectives of building country preparedness for future outbreaks while contributing to other health areas. The insights from this supplement illustrate that strategically investing in the digital health enabling environment—including country-level digital health governance and workforce capacity, as well as in interoperable digital enterprise architecture—delivers a multiplying effect, both supporting global health security and helping emergency investments outlive the crisis phase to improve country resilience.
